# Cancer Antigen 125 during Pregnancy in Women without Ovarian Tumor Is Not Often Rising

**DOI:** 10.1155/2018/8141583

**Published:** 2018-04-01

**Authors:** Rattapon Amampai, Prapaporn Suprasert

**Affiliations:** Department of Obstetrics and Gynecology, Faculty of Medicine, Chiang Mai University, Chiang Mai 50200, Thailand

## Abstract

**Objective:**

To determine the percentage of rising serum cancer antigen (CA-125) in singleton pregnant women whose ultrasonographical findings were normal.

**Methods:**

Singleton pregnant women who received antenatal care at our institute with a normal ultrasonographical examination in their first and/or second trimester were invited to participate in blood testing for CA-125. The conditions that might affect the CA-125 level were excluded. The normal level of CA-125 was defined as ≤35 U/ml.

**Results:**

136 pregnant women met the inclusion criteria. Of these cases, 87 cases received a blood test for CA-125 in both their first and second trimesters while 46 and 3 cases received a blood test for CA-125 in only the first and second trimester, respectively. The median serum CA-125 levels in the first and second trimester were 16.44 (range 5.94–77.54) U/ml and 16.76 (range 5.26–35.81) U/ml, respectively. Only 9.1% of the studied patients showed an abnormal CA-125 level in the first trimester period and only one case showed an abnormal CA-125 level in the second trimester period.

**Conclusion:**

Few of normal pregnancies showed rising CA-125. Therefore, when it elevated in pregnant women, other causes such as the adnexal lesion should be investigated.

## 1. Introduction

Nowadays, in an era of routine prenatal ultrasonography, ovarian tumors have been detected in up to two percent of pregnant women. Only two to three percent of these ovarian tumors were ovarian cancer that needed to have specific management including complete surgical staging and chemotherapy [1]. Cancer antigen 125 (CA-125) is a biomarker usually expressed at the atypical surface of the coelomic epithelium. The rising of serum CA-125 more than 35 U/ml has been shown to enhance the potential of malignancy in those ovarian tumors. Thus, the measurement of serum CA-125 is still commonly used as the index of ovarian cancer even though a variety of benign gynecological and nongynecological conditions also increase the level of CA-125 including endometriosis, functional ovarian cysts, pelvic inflammation, cirrhosis, colitis, renal disease, tuberculosis, and pregnancy [2, 3].

Regarding pregnancy, fetal chorion, amniotic fluid, and maternal decidua have also been shown to contain significant amounts of CA-125 and may represent potential sources of elevation of serum CA-125 [4]. However, the rising of CA-125 in normal pregnancy is still unclear. Some previous publications did not find any abnormal rising of CA-125 [2, 5] while some publications showed high levels of CA-125 in normal pregnancy [6, 7]. To find out more data about the level of serum CA-125 in normal pregnancy, this study was conducted to determine the percentage of abnormal rising of serum CA-125 in singleton pregnant women whose prenatal ultrasounds were unremarkable.

## 2. Materials and Methods

This prospective study was conducted after approval from the Ethics Committee of the Faculty of Medicine, Chiang Mai University. The inclusion criteria included first and second trimester Thai singleton pregnant women defined as having a Thai identification card, no underlying disease, and no history of cancer or endometriosis who attended the Antenatal Care Unit at Maharaj Nakorn Chiang Mai Hospital. The first trimester gestational age was less than or equal to 12 weeks, and the second trimester was the gestational age of 13–25 weeks. Pregnant women with abnormal ultrasound findings of the uterus, ovary, or fetus were excluded. After informed consent, the blood sample for CA-125 testing was collected in the first and/or second trimester of pregnancy and examined at the laboratory of Maharaj Nakorn Chiang Mai Hospital using Elecsys® analyzer and CA-125 II reagents (Roche Diagnostics) with the reference range of 0–35 U/ml [8].

The sample size for this trial was estimated on basis of a prior study that revealed 95% of pregnant women showing normal CA-125 levels [2]. The 95 interpercentile reference intervals for calculation accommodated the possibility to loss of follow up participants. Finally, this study needed to enroll 130 participants.

The clinical data including maternal demographics, prepregnancy health, and pregnancy characteristics were recorded. Statistical analysis was performed by SPSS version 21.0. The descriptive data were presented as mean ± SD or median as appropriate.

## 3. Results

**Figure 1:**
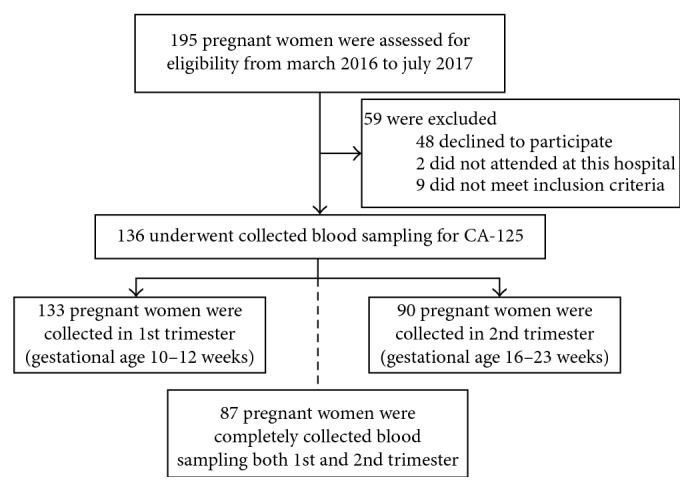
Flow of participants through the study.

Between March 2016 and July 2017, a total of 195 pregnant women were invited to participate in this study. However, 59 women were excluded due to declining to participate (48), being attended at other hospital (2), foreigner (2), blighted ovum (1), early fetal death (2), history of myoma (1), history of endometriosis (1), history of ovarian tumor (1), and history of kidney disease (1). The remaining 136 women met the inclusion criteria and participated in the present study. Of these participants, 87 pregnant women received a blood test for serum CA-125 in both the first and second trimesters while 46 and three patients received a test for serum CA-125 in only first and second trimester, respectively, as shown in Figure 1.

The baseline characteristics of the participants are listed in Table 1. The mean age was 29.53 years old and the mean BMI was 21.9 kg/m^2^. Most of the participants graduated from senior high school and held a bachelor's degree. About 60% were nulliparous and nearly 30% experienced an abortion.

**Figure 2:**
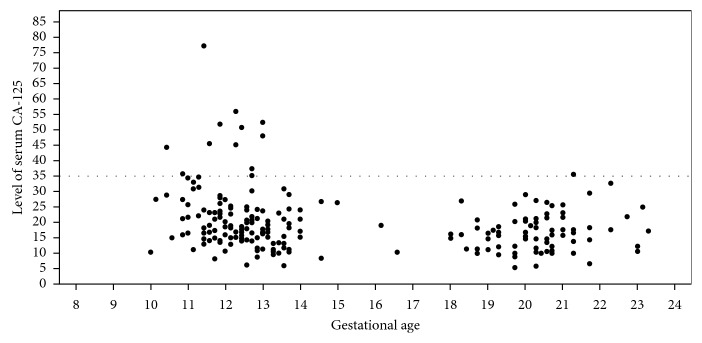
The distribution of serum CA-125 in each gestational age.

Regarding serum CA-125 level, the distribution of the value of serum CA-125 in each gestational age is presented in Figure 2. The median level of serum CA-125 level in the first (gestational age 10–12 weeks) and second trimesters (gestational age 16–23 weeks) was 16.44 (range 5.94–77.54) U/ml and 16.76 (range 5.26–35.81) U/ml, respectively. Of those 133 participants' serum CA-125 in the first trimester period, only 12 cases (9.02%) showed abnormal rising level of CA-125 while only one from 90 cases (1.11%) in the second trimester period showed an abnormal serum CA-125 level. About 12 cases whose CA-125 were abnormal in the first trimester, 7 cases were measured CA-125 level in the second trimester period and showed minimal rising level of CA-125 as 35.81 U/ml in 1 case while the remaining were normal. In addition, the patient with the highest level of serum CA-125 (77.54 U/ml) was missed for collection of the second trimester blood test for CA-125. However, she had no uneventful obstetric outcomes.

## 4. Discussion

Based on our findings, over 90% of Thai pregnant women who had no ultrasonographical evidence of ovarian or uterine tumor and no other possible causes of rising CA-125 showed a normal serum CA-125 value in the first and second trimesters. Only 9.02% of these pregnant women had a level of serum CA-125 more than 35 U/ml in the first trimester period. Of those cases with an abnormal level of CA-125, their CA-125 level declined to a normal level except in one case that continued to be higher than the normal range in the second trimester period.

Regarding previous studies, there were different outcomes of serum CA-125 levels in pregnancy. The largest series was from Denmark [2] in which the authors studied serum CA-125 levels in 720 Caucasian pregnant women with 4,574 samples obtained at various stages of pregnancy including gestational weeks 13–20, 21–28, 29–34, 35–42, at delivery, and in the first two postpartum days. The results showed that serum CA-125 levels were stable and remained below the conventional cut-off level at 35 U/ml during pregnancy. However, CA-125 was rising at delivery and in the postpartum period. They suggested that CA-125 cut-off value less than 35 U/ml used for nonpregnant women could be used for women with pregnancy after gestational age 13 weeks as additional data when ovarian tumor was an incidental finding at that time. In contrast to Aslam et al. [9], measuring serum CA-125 at 11–14 weeks of gestation in 188 women with morphologically normal ovaries found the median serum CA-125 was 23.4 U/ml with a range of 2.2–166.3 U/ml, with about 20% findings higher than 35 U/mL. This percentage was higher than the present study that found 9% of the Thai pregnant women with an abnormal level of CA-125 in the first trimester. Another study measured serum CA-125 in a small number of pregnant Japanese women (29 cases in first trimester and 21 cases in second and third trimesters) without mentioning the adnexal morphology. They reported mean levels of CA-125 very high at 85 U/ml in first trimester but only 20 U/ml and 25 U/ml in the second and third trimesters, respectively [6]. An Iraqi study measured serum CA-125 in 24 normal intrauterine pregnancies between 4 and 10 weeks' gestational age with the mean ± SD level of serum CA-125 of 74.25 ± 18.5 U/ml with a range of 28–200 U/ml. This was significantly higher than levels of serum CA in 40 ectopic pregnant that showed the mean ± SD of serum CA-125 only 16.51 ± 2.39 U/ml (2–80 U/ml) [7]. The variable of serum CA-125 levels in different publications including the present study might be due to different ethnicity, the inclusion and exclusion criteria, and the number of studied participants. However, CA-125 level in first trimester was higher than second trimester in most results. For the third trimester, Ozat et al. found rising CA-125 in women with preeclampsia and reported high sensitivity and specificity as 93.7 and 88%, respectively, when using a cut-off point of serum CA-125 at 50 U/ml to predict obstetric outcomes [10]. Recently, Nan et al. published the systematic review about the physiologic variations of serum tumor markers in gynecological malignancies during pregnancy and summarized the outcome form 10 publications about serum CA-125 level. The result found that all of them revealed elevated serum CA-125 level up to 35% of the measurements and uniformly reported to be the highest in the first trimester with the maximum value up to 550 U/mL [11] This percentage was higher than our study that found only 10% showed rising level. However, our study was confirmed the normal pregnancy status by ultrasonography while some studies in those Nan's study did not.

The strengths of the present study were that all participants had confirmed adnexal status by expert ultrasonologists with other possible causes of rising serum CA-125 excluded from the study. Therefore, the results represented CA-125 levels from normal pregnant women. Besides this, most participants received serum CA-125 testing in both the first and second trimester periods which represented the trend of serum CA-125 in the same population. However, about one-third of these participants were lost for blood collection test for CA-125 in the second trimester including one case with the highest level of CA-125 in this study (77.54 U/ml). Thus, we did not know whether or not this case still had a high level of CA-125 in her second trimester period. She had no eventful obstetric outcomes.

In conclusion, for normal pregnant women, the serum CA-125 level was mostly within the normal range. Only 10% of them revealed rising of CA-125. Hence, the rising of serum CA-125 in those pregnant women with ovarian tumors should be of concern as a possible cause besides pregnancy.

## Figures and Tables

**Table 1:** Basic clinical data (*N* = 136).

	*N* (%)
Mean age (±SD), year	29.53 (±4.47)
Mean BMI (kg/m^2^)	21.90 (4.10)
Education	
Primary school	2 (1.5)
Junior high school	9 (6.6)
Senior high school/vocational school	58 (42.3)
Bachelor's degree	60 (44.1)
Master's degree	7 (5.1)
Gravida	
1	55 (40.4)
2	52 (38.2)
3	22 (16.2)
4	4 (2.9)
5	2 (1.5)
6	1 (0.7)
Parity	
Nulliparity	80 (58.8)
Multiparity	56 (41.2)
Abortion	
None	91 (66.9)
1	36 (26.5)
2	8 (5.9)
4	1 (0.7)
Curettage	
None	112 (82.4)
1	19 (14.0)
2	5 (3.7)
